# Impact of history of mental disorders on short-term mortality among hospitalized patients with sepsis: A population-based cohort study

**DOI:** 10.1371/journal.pone.0265240

**Published:** 2022-03-10

**Authors:** Lavi Oud, John Garza

**Affiliations:** 1 Division of Pulmonary and Critical Care Medicine, Department of Internal Medicine, Texas Tech University Health Sciences Center at the Permian Basin, Odessa, Texas, United States of America; 2 Department of Mathematics, The University of Texas Permian Basin, Odessa, Texas, United States of America; Wayne State University, UNITED STATES

## Abstract

**Background:**

Mental disorders are associated with markedly reduced life expectancy, in part due to an increased risk of death due to infection, likely reflecting sepsis-associated mortality. Patients with mental disorders are at an increased risk of sepsis, but data on the prognostic impact of mental disorders in sepsis are sparse, showing conflicting findings.

**Methods:**

We used statewide data to identify hospitalizations aged ≥18 years with sepsis in Texas during 2014–2017. Mental disorders, including mood, anxiety, psychosis, and personality disorders were identified using Clinical Classification Software codes. Multilevel, multivariable logistic regression with propensity adjustment (primary model), with propensity score matching, and multivariable logistic regression as alternative models, were used to estimate the association between mental disorders and short-term mortality (defined as in-hospital mortality or discharge to hospice). Additional models were fitted for sensitivity analyses and to estimate the prognostic associations of individual categories of mental disorders.

**Results:**

Among 283,025 hospitalizations with sepsis, 56,904 (20.1%) had mental disorders. Hospitalizations with vs without mental disorders were younger (age 18–44 years 12.2% vs 10.6%), more commonly white (61.0% vs 49.8%), with lower burden of comorbidities (mean [SD] Deyo comorbidity index 2.53 [2.27] vs 2.73 [2.47]), and with lower need for organ support (mechanical ventilation 32.8% vs 36.0%); p<0.0001 for all comparisons. Crude short-term mortality among sepsis hospitalizations with and without mental disorders was 25.0% vs 32.8%, respectively. On adjusted analyses, mental disorders remained associated with lower odds of short-term mortality (adjusted odds ratio 0.792 [95% CI 0.772–0.812]). This finding was consistent on the alternative modeling approaches, sensitivity analyses, and examination of individual categories of mental disorders.

**Conclusions:**

Mental disorders were associated, unexpectedly, with markedly lower risk of short-term mortality in sepsis. Further studies to examine the mechanisms underlying these findings may inform future efforts to improve sepsis outcomes.

## Introduction

Sepsis remains a major global health burden with estimated nearly 49 million cases worldwide in 2017 and 11 million sepsis-related deaths [1]. Despite improving sepsis-associated mortality over the past decades [2, 3], attributed in part to care improvements, considerable outcome disparities remain among affected patients [1, 4, 5]. A more comprehensive understanding of the conditions associated with increased mortality in septic patients can inform targeted policy- and clinical practice-related preventive and interventional efforts. Contemporary efforts to improve sepsis outcomes have focused mostly on outcome disparities associated with sociodemographic traits (e.g., race [4]) and pre-existing “physical” illness (e.g., cancer [5]).

Mental disorders are highly prevalent, estimated to affect over 970 million people worldwide in 2017 [6], and are increasingly recognized as the major contributor to years lived with disability [7]. Crucially, mental disorders shorten life expectancy by 11–20 years [8], primarily due to natural causes, with 2.2-fold higher risk of all-cause mortality compared to the general population [9], resulting in approximately 8 million attributable deaths per year worldwide [9]. The mechanisms underlying the shortened life expectancy in affected patients are not completely understood, but the increased risk of mortality is thought to be related to prevalent risky lifestyle choices, altered engagement with the health care system, increased risk of comorbid conditions, and poorer quality of preventive and interventional medical care [10]. In 2019, only 44.8% of adults with mental disorders in the United States received mental health services within the preceding 12 months [11], with markedly lower rates in lower-resource countries [12].

Infections are one of the causes of reduced life expectancy in patients with mental disorders, with 1.9 to 5.2-times higher risk of death due to an infection, compared to the general population [8], with sepsis considered the major cause of death from infectious diseases [1, 13]. The increased risk of infection-related deaths among patients with mental disorders may represent higher risk of infection and sepsis, increased case fatality among septic patients, or a combination of both. Distinction between these possibilities is important, as it would guide different interventions. Numerous studies have linked mental disorders with increased risk of infection [14–17] and sepsis [15, 17, 18]. On the other hand, data on the prognostic impact of mental disorders in sepsis are sparse and show mixed findings.

Two recent population-based reports from Denmark, studying outcomes of hospitalizations with an infection found increased 30-day mortality among the subgroup with sepsis among patients who had mental disorders, compared to those without mental disorders [19, 20]. Although the specific causes of the poorer outcomes among septic patients with mental disorders were unclear, the investigators hypothesized that poor self-care, lower adherence to therapy of chronic medical conditions, higher burden of physical comorbidities, and differences in preventive and inpatient care may have played a role [19, 20]. On the other hand, in three other population-based studies from Germany [21] and the United States [22, 23] that sought to examine potential prognostic factors in severe sepsis and septic shock, presence of depression or psychosis were each associated with lower risk of death. None of these latter studies focused specifically on the findings related to mental disorders. The sources of the conflicting findings between these studies are unclear. In addition, comparisons between these reports and the generalizability of their findings are further constrained by lack of reported data on the characteristics and mortality rates of septic patients with and without mental disorders in these studies [19–23].

Because mental disorders remain a major global health burden, a better understanding of their prognostic role in sepsis can inform health policy, resource allocation, and targeted efforts to improve sepsis outcomes in this population. Here, we report a population-based study of adult hospitalizations with sepsis, seeking to examine the association of mental disorders with short-term mortality. We hypothesized that mental disorders are associated with increased risk of death.

## Materials and methods

### Study design and data sources

We performed a retrospective, population-based cohort study using the Texas Inpatient Public Use Data File (TIPUDF). The TIPUDF is a publicly available, de-identified dataset, and the study was determined to be exempt from formal review by the Texas Tech Health Sciences Center Institutional Review Board. Several components of the study approach and data analyses were previously reported [24]. The reporting of the study follows the Strengthening the Reporting of Observational Studies in Epidemiology (STROBE) guidelines [25].

The TIPUDF is an all-payer administrative dataset maintained by the Texas Department of State Health Services [26]. The dataset includes information from non-federal hospitals on patients’ demographics, diagnoses and procedures, and hospital disposition, capturing approximately 97% of all hospital discharges in the state. We used hospitalizations as the unit of analysis, instead of patients, since the TIPUDF dataset provides discharge-level, rather than patient-level information, which precludes accounting for repeated admissions.

### Patients and variables

#### Cohort derivation

Our primary cohort consisted of hospitalizations aged ≥18 years with a diagnosis of sepsis during the years 2014–2017. We excluded hospitalizations with missing data on hospital disposition. We identified hospitalizations with sepsis based on the presence of the *International Classification of Diseases*, *Ninth and Tenth Revisions*, *Clinical Modification (ICD-9-CM and ICD-10-CM*, *respectively)* codes for severe sepsis (995.92, R65.20) and septic shock (785.52, R65.21) under the principal or secondary diagnosis fields. This ICD code-based definition of sepsis is aligned with the framework of Sepsis-3 [13] and has been used in contemporary studies of sepsis in administrative data [7, 8, 27]. Hospitalizations with ICU admissions were identified based on unit-specific revenue codes for an intensive care unit or a coronary care unit.

#### Exposure and outcome

The primary exposure was a diagnosis of a mental disorder. Hospitalizations with mental disorders were identified by the presence of specific Healthcare Cost and Utilization Project’s Clinical Classification Software (CCS) codes [28]. CCS codes incorporate multiple associated ICD-9-CM and ICD-10-CM codes into smaller, more clinically meaningful categories. We used CCS codes 651 (anxiety disorders), 657 (mood disorders), 658 (personality disorders), or 659 (schizophrenia and other psychotic disorders) to derive the subgroup with mental disorders. Similar categorization of mental disorders has been used in other epidemiological studies [29, 30]. The primary outcome was short-term mortality, defined as in-hospital death or discharge to hospice.

#### Risk-adjustment covariates

Risk-adjustment covariates were selected *a priori* based on biological and clinical plausibility and existing literature [5, 31, 32] and included patients’ demographics (age, gender, race/ethnicity, primary health insurance), major comorbidities (based on the Deyo modification of the Charlson Comorbidity Index [33, 34]), alcohol use disorders (CCS category 660) [35], substance use disorders (CCS category 661) [22], hospitals’ teaching status [36], and year of admission [37]. Severity of illness was characterized using ICD codes for organ dysfunction as defined by Martin and colleagues [38]. Procedure use was identified using ICD-9 and ICD-10 procedure codes for mechanical ventilation, hemodialysis, and blood transfusion (S1 Table in S1 File).

### Statistical analysis

We summarized categorical variables as frequencies and percentages, while continuous variables were reported as mean and standard deviation. The chi-square test was used for group comparison involving categorical variables, while the t-test was used for comparison of continuous variables.

We used three distinct prespecified analytical approaches to examine the association between a diagnosis of mental disorder and short-term mortality: 1) multilevel multivariable logistic regression with propensity adjustment (our primary analysis) 2) propensity score matching and 3) multivariable logistic regression without propensity adjustment. Only the primary analysis (multilevel multivariable regression with propensity adjustment) was performed for individual categories of mental disorders, subgroups, and sensitivity analyses. Multicollinearity was excluded using variance inflation factors. The Box-Tidwell test was used to confirm assumption of linearity of continuous risk-adjustment covariates.

#### Propensity score calculation

A propensity score is a probability-based measure indicating the propensity of a septic patient to have a mental disorder. We calculated the propensity for mental disorder among septic hospitalizations using multivariable logistic regression with mental disorder used as the dependent variable. The variables included in the model were: age, gender, race/ethnicity, primary health insurance, Deyo comorbidity index, congestive heart failure, chronic lung disease, cerebrovascular disease, diabetes, chronic renal disease, liver disease, malignancy, alcohol use disorders, and substance use disorders.

#### Multilevel multivariable logistic regression with propensity adjustment

We used multilevel multivariable logistic regression to assess the association between mental disorders and short-term mortality in septic hospitalizations. The covariates entered into the multivariate model included all those described for risk-adjustment, as well as mental disorders, and with individual hospitals entered as random intercepts to account for clustering of hospitalizations within hospitals. The propensity score was added as an independent variable. Combining regression analysis and propensity scores into a single model, known as double robust estimation [39], allows all study patients to be retained in the analysis, as opposed to traditional 1:1 propensity score matching, which excludes unmatched patients and thus can reduce model’s generalizability. In addition, this method provides more accurate estimation of variance and reduces bias [40].

#### Propensity score matching

Hospitalizations with mental disorders were matched to those without mental disorders, but had similar propensity for them. Matching was performed with 1:1 nearest neighbor matching without replacement, using maximal caliper width equal to 20% of the standard deviation of the logit of the propensity score [41]. We examined the balance of each covariate between the matched cohorts with and without mental disorders using standardized differences and histograms, without consideration of any outcome variable. A standardized difference <0.1 was considered to represent well-balanced covariates [42]. We re-matched the comparison cohorts if any covariates were unbalanced on the initial match and then re-examined each covariate to ensure adequate balance between the comparison cohorts with and without mental disorders before examining any results.

#### Multivariable logistic regression without propensity adjustment

We used multivariable logistic regression to assess the association between mental disorders and short-term mortality, using the same covariates used, as described for our primary analysis.

The State of Texas masks gender data of hospitalizations with a diagnosis of HIV infection, and of those with ethanol or drug abuse. Gender data were missing nonrandomly in 8.8% of hospitalizations in our cohort. We examined the sensitivity of the association between mental disorders and short-term mortality to missing gender data using three approaches. The details of the alternative modeling approaches are provided in the Supporting information file and the model results, which produced similar findings for each of the three models, are available in S2-S4 Tables in S1 File. In this article, we present the results of our analyses using data restricted to hospitalizations with gender data. We report the models’ findings as adjusted odds ratios (aOR) and 95% confidence intervals (95% CI).

#### Individual categories of mental disorders and short-term mortality

We performed additional analyses to examine the association between the specific categories of mental disorders included in our cohort and short-term mortality. The primary analysis approach was used for these analyses.

#### Subgroup analyses

Exploratory analyses were performed to examine the consistency of the association between mental disorders and short-term mortality among *a priori* selected subgroups, including age, gender, race/ethnicity, and health insurance. The later subgroup was chosen as a proxy of socioeconomic status. The primary analysis approach was used for subgroup analyses.

#### Sensitivity analyses

In order to further assess the robustness of the observed association between mental disorders and short-term mortality we performed sensitivity analyses restricted to the more severe septic hospitalizations, including those admitted to ICU and those with septic shock. The primary analysis approach was used for sensitivity analyses.

Data management was performed using Microsoft Excel (Microsoft, Redmond, Washington) and statistical analyses were performed with R 3.6.0 (R Foundation for Statistical Computing, Vienna, Austria). The R code supporting these analyses is provided in the Supporting information file. A 2-sided *p* value < 0.05 was considered statistically significant.

## Results

We identified 283,025 hospitalizations with sepsis, of which 56,904 (20.1%) had a mental disorder. Propensity score matching yielded 50,174 pairs of sepsis hospitalizations with and without mental disorders. All patient characteristics modeled for the propensity score were statistically balanced (S5 Table in S1 File). The histogram display of the pre- and post- match groups is provided in S1 Fig in S1 File.

### Cohort characteristics

The characteristics of sepsis hospitalizations with and without mental disorders for the whole cohort are detailed in Table 1. Compared to sepsis hospitalizations without mental disorders, those with a mental disorder were younger (aged 18–44 years: 12.2% vs. 10.6%, respectively), more commonly white (61.0% vs 49.8%, respectively), with a lower burden of comorbidities (mean Deyo comorbidity index 2.53 vs 2.73, respectively), and with lower need for organ support (use of mechanical ventilation 32.8% vs. 36.0%, respectively). Among sepsis hospitalizations with mental disorders, mood (66.0%) and anxiety (48.4%) disorders were the most common categories.

**Table 1:** The characteristics of septic hospitalizations with and without mental disorders.

Variables	Mental disorders^a^	No mental disorders^a^	*p* value
	n = 56,904	n = 226,121	
**Age, years**			<0.0001
18–44	6,918 (12.2)	23,939 (10.6)	
45–64	21,301 (37.4)	70,412 (45.5)	
≥ 65	28,685 (50.4)	131,770 (58.3)	
**Gender** ^b^	30,857 (61.3)	131,770 (48.1)	<0.0001
Female	1,878 (49.5)	99,255 (47.8)	
**Race/ethnicity**			<0.0001
White	34,720 (61.0)	112,499 (49.8)	
Hispanic	12,365 (21.7)	63,117 (27.9)	
Black	5,979 (10.5)	31,061 (13.7)	
Other	3,832 (6.7)	19,390 (8.6)	
**Health insurance** ^c^			<0.0001
Private	17,776 (31.2)	74,688 (33.0)	
Medicare	29,121 (51.2)	112,053 (49.6)	
Medicaid	5,259 (9.2)	15,827 (7.0)	
Uninsured	3,953 (6.9)	20,398 (9.0)	
Other	707 (1.2)	2,897 (1.3)	
**Deyo comorbidity index** (mean, SD^d^)	2.53 (2.27)	2.72 (2.47)	<0.0001
**Major comorbidities**			
Chronic lung disease	18,928 (33.3)	56,652 (25.0)	<0.0001
Congestive heart failure	16,515 (29.0)	71,271 (31.5)	<0.0001
Cerebrovascular disease	4,693 (8.2)	21,494 (9.5)	<0.0001
Renal disease	16,279 (28.6)	78,207 (34.6)	<0.0001
Diabetes	21,460 (37.7)	91,612 (40.5)	<0.0001
Malignancy	8,598 (10.4)	33,895 (15.0)	<0.0001
Liver disease	5,829 (10.2)	27,389 (12.1)	<0.0001
**Alcohol use disorders**	3,904 (6.9)	14,597 (6.5)	0.0006
**Substance use disorders**	11,209 (19.7)	27,887 (12.3)	<0.0001
**Categories of mental disorders**		NA	
Anxiety	27,529 (48.4)		
Mood	37,574 (66.0)		
Personality	358 (0.6)		
Psychosis	5,961 (10.5)		
**Number of organ failures** (mean, SD^d^)	2.52 (1.42)	2.73 (1.51)	<0.0001
**Type of organ failures**			
Respiratory	31,202 (54.8)	126,943 (56.1)	<0.0001
Cardiovascular	34,718 (61.0)	147,137 (65.0)	<0.0001
Renal	32,686 (57.4)	140,282 (62.0)	<0.0001
Hepatic	3,784 (6.6)	21,177 (9.4)	<0.0001
Hematological	9,920 (17.4)	49,069 (21.7)	<0.0001
Neurological	16,234 (28.5)	62,187 (27.5)	<0.0001
**Mechanical ventilation**	18,678 (32.8)	81,564 (36.0)	<0.0001
**Hemodialysis**	5,422 (9.5)	28,659 (12.7)	<0.0001
**Blood transfusion**	9,974 (17.5)	46,534 (20.6)	<0.0001
**Teaching hospital**	15,528 (27.2)	63,422 (28.0)	<0.0001
**Hospital disposition**			
Hospital mortality	9,957 (17.5)	55,480 (24.5)	<0.0001
Hospice	4,251 (7.5)	18,787 (8.3)	<0.0001
Home	20,877 (36.7)	80,255 (35.5)	<0.0001
Another acute care hospital	2,230 (3.9)	8,061 (3.6)	<0.0001
Chronic care facility^e^	19,005 (33.4)	62,013 (27.4)	0.0001
Leave against medical advise	584 (1.0)	1,525 (0.7)	<0.0001

^a^The parenthesized figures represent percents, except for Deyo comorbidity index and number of organ failures;

Percentage figures may not add to 100 due to rounding

^b^Gender was missing for 6559 hospitalizations with mental disorders and for 18435 hospitalizations without mental disorders;

the percent figures for gender in each column refer to that column’s denominator for gender

^c^Health insurance data were missing for 88 hospitalizations with mental disorders and for 258 hospitalizations without mental disorders

^d^SD: Standard deviation

^e^Chronic care facilities include: long-term care hospitals, inpatient rehabilitation, skilled nursing facilities, and nursing homes

### The impact of mental disorders on short-term mortality

The details of hospital disposition of cohort hospitalizations are provided in Table 1. Sepsis hospitalizations with mental disorders had substantially lower crude short-term mortality, compared to those without mental disorders (25.0% vs. 32.8%, respectively). The reduced risk of short-term mortality among sepsis hospitalizations with a mental disorder remained on adjusted analyses of the primary model, being 21% lower (aOR 0.792 [95% CI 0.772–0.812]), with similar findings on the alternative modeling analyses (Table 2). Each of the examined categories of mental disorders was associated with lower odds of short-term mortality among sepsis hospitalizations (Table 3).

**Table 2:** Odds ratios for short-term mortality associated with mental disorders.

Model	aOR (95% CI)^a^	*p* value
Multilevel multivariable logistic regression with propensity adjustment	0.7924 (0.7728–0.8125)	<0.0001
Alternative analyses		
Propensity score-matched sample	0.7968 (0.7688–0.8257)	<0.0001
Multivariable logistic regression without propensity adjustment	0.8062 (0.7865–0.8264)	<0.0001

^a^aOR (95% CI): adjusted odds ratio and 95% confidence intervals

**Table 3:** Odds ratios for short-term mortality associated with specific categories of mental disorders^a^.

Category	aOR (95% CI)^b^	*p* value
Anxiety disorders	0.8325 (0.8046–0.8613)	<0.0001
Mood disorders	0.7781 (0.7552–0.8017)	<0.0001
Personality disorders	0.4509 (0.3047–0.6671)	<0.0001
Psychotic disorders	0.7398 (0.6889–0.7944)	<0.0001

^a^all analyses were carried out using multilevel multivariable logistic regression with propensity adjustment

^b^aOR (95% CI): adjusted odds ratio and 95% confidence intervals

**Fig 1:**
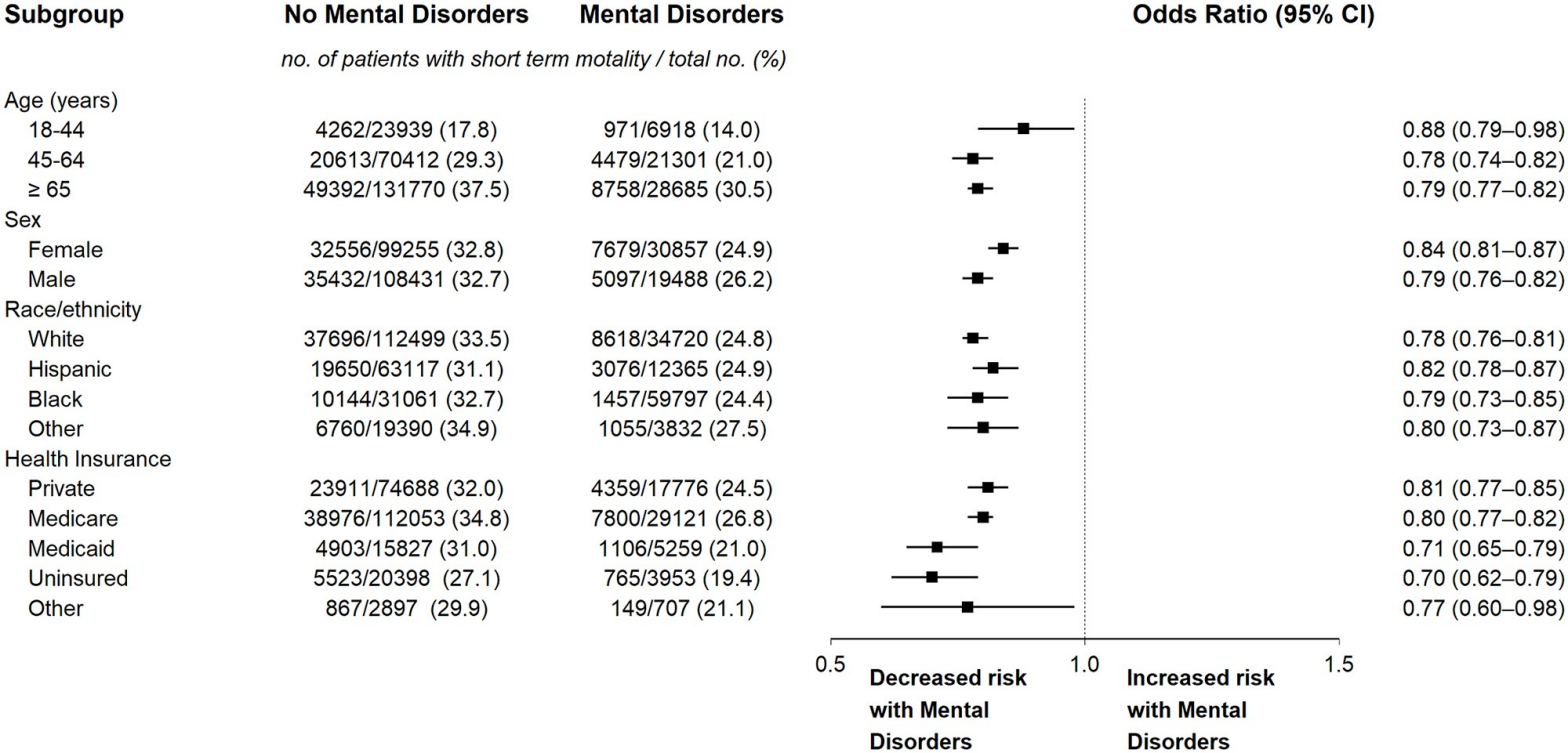
Subgroup analyses of short-term mortality of septic hospitalizations with and without mental disorders. Analyses were done using multilevel multivariable logistic regression with propensity adjustment. The odds ratios and 95% confidence intervals have not been adjusted for multiplicity and should not be used to infer definite effects.

The findings on subgroup analyses of the association of mental disorders with short-term mortality are detailed in Fig 1. Mental disorders were associated with lower risk of short-term mortality among sepsis hospitalizations in each of the examined subgroups.

Sensitivity analyses showed similar findings of the association of mental disorders with short-term mortality as the primary model among sepsis hospitalizations admitted to ICU (aOR 0.797 [95% CI 0.769–0.826]) and those with septic shock (aOR 0.762 [(95% CI 0.7390.785]).

## Discussion

### Key findings

In this population-based study, presence of mental disorders among patients with sepsis was associated with 21% lower risk of short-term mortality. This finding was robust across 3 distinct modeling approaches, as well as within subgroup analyses, with similarly consistent findings within individual categories of mental disorders.

### Relationship to previous studies

It was our hypothesis that the many health-related challenges faced by patients with mental disorders, outlined earlier, will increase their risk of death following sepsis. However, our analyses showed, unexpectedly, that presence of mental disorders was associated with reduced risk of short-term mortality in a large sepsis cohort. Although our findings are concordant with other studies from Germany [21] and the US [22, 23], more detailed comparisons are precluded by lack of data on the characteristics of septic patients with and without mental disorders in these reports.

The sources of the contrasting findings between our cohort and the increased short-term mortality among septic patients with mental disorders in the studies from Denmark are unclear. The Danish studies reported only the characteristics of infected hospitalized patients with and without mental disorders, but not those of the subgroup with sepsis [19, 20]. Infected patients with mental disorders in these latter studies were older and had higher burden of chronic illness than those without these conditions. It is unlikely that the subgroup of septic patients with mental disorders in these studies were younger and healthier than those with infections in general. By contrast, the favorable outcomes among patients with mental disorders in our study could be explained by their younger age and lower comorbidity burden compared to those without mental disorders, as well as by their higher frequency of white race, as racial and ethnic minority status is associated with higher mortality in sepsis [4]. However, the lower risk of short-term mortality among sepsis hospitalizations with mental disorders in our cohort remained following addressing these groups imbalances on risk-adjusted analyses. On the other hand, although the increased risk of short-term mortality among septic patients with mental disorders in the Danish studies remained on adjusted analyses, the investigators did not report on, nor adjust their analyses for measures of illness severity or organ support [19, 20], which could further explain the observed outcome differences across studies. Nevertheless, we cannot exclude the possibility that differences between the populations of US and Denmark beyond those captured in cohort characteristics and analytical methods may have contributed to the differences in the prognostic impact of mental disorders between our cohort and the Danish studies.

Notably, the presence of more favorable demographic and clinical characteristics among patients with mental disorders was not associated with more favorable outcomes in studies that did not focus on sepsis. In two recent studies that evaluated the prognostic impact of mental disorders in a heterogenous population of medical and surgical critically ill patients in the US, the groups with mental disorders were, similarly to our cohort, younger, more commonly white, with lower burden of comorbidities, and had lower illness severity [43, 44]. However, on adjusted analyses, mental disorders were associated with either no prognostic effect [43] or higher 30-day mortality [44]. Indeed, in numerous other studies of the prognostic implications of mental disorders in hospitalized patients where sepsis was not the condition of interest, presence of mental disorders had no prognostic advantage, but was rather associated with higher risk of death [45–49].

The mechanisms underlying the lower risk of short-term mortality associated with mental disorders among septic patients are unclear. Two potential contributors may be considered for the favorable outcome associations observed in the present study and in prior reports.

First, the response to infection could have differed in patients with mental disorders who progressed to sepsis, compared to the general population. Several disparate data lines may be linked in the examination of this postulate. Over the past two decades mental disorders were increasingly shown to be associated with immune dysfunction, involving the activation of innate and adaptive cellular effectors [50–52], which is hypothesized to play a causal role in the pathogenesis and clinical expression of these disorders [53–56]. Numerous studies demonstrated systemic low-grade chronic inflammation profile in depression, bipolar disorders, schizophrenia, and in anxiety and personality disorders, characterized by increases in peripheral levels of interleukin-1 (IL)-1 [54, 55, 57–59], IL-6 [54, 55, 57–59], IL-12 [54, 60, 61], interferon-gamma (IFN-γ) [54, 55, 57, 62], and tumor necrosis factor-alpha (TNF-α) [54, 55, 57–59, 61], and decreased levels of IL-4 [54, 58] and IL-10 [54, 55, 62].

Second, recent studies have shown an increased risk of autoimmune disease among patients with depression [63], bipolar disorders [64], schizophrenia [64] and post-traumatic stress disorder [65]. Although the mechanisms for the increased risk of autoimmunity in these populations have not been elucidated, it has been hypothesized that their dysregulated immune state may underlie these observations [63, 65].

Finally, although patients with autoimmune diseases are at an increased risk of infections and sepsis, a recent study by Sheth et al found that among septic patients, those with autoimmune diseases had markedly lower 30-day mortality [66]. On further adjusted analyses, stratified by the level of expression of specific cytokines in autoimmune diseases, 30-day mortality was reduced in those autoimmune diseases with overexpression of IL-1, IL-6, IL-12, IFN-γ, and TNF-α, as well as in those with reduced expression of IL-4 and IL-10 [66]. These associations between pre-sepsis immune dysfunction and mortality are supported by studies showing that sepsis impairs production of IL-1, IL-6, TNF-α [67], IL-12 [68], and IFN-γ [69] and suggesting that this sepsis-induced immunosuppressive state may be augmented by release of IL-4 and IL-10 [69]. The authors hypothesized [66] that patients with pre-sepsis over- and under-expression of specific cytokines may be better suited to survive sepsis-induced impairment in immune function [69–72].

Together, the abovementioned data suggest possible difference in response to infection between those with and without mental disorders, related to the baseline immune dysfunction of the former. However, despite the compelling similarities between the prognostic associations noted in the study by Sheth et al [66] and the cytokine profiles reported in patients with mental disorders, no direct comparative studies of the immune responses of septic patients with and without mental disorders (or those with vs without autoimmune disease) were performed to date. Thus, further studies are needed to both corroborate our findings and to examine sepsis-associated changes across immune function domains in patients with and without mental disorders to further inform strategies to improve sepsis outcomes.

Another potential contributor to reduced short-term mortality among septic patients with mental disorders may be related to the immunomodulating effects of psychotropic agents. Immunomodulating properties of these agents were noted in both *in vitro* and *in vivo* studies, though the effects varied across agents and studies [72, 73]. Relatively limited preclinical evidence suggests potentially beneficial effects of psychotropic agents in sepsis. A study of a murine model of septic shock demonstrated decreased mortality following treatment with fluvoxamine [74]. Fluvoxamine is a selective serotonin reuptake inhibitor and a strong agonist of sigma-1-receptor, a resident chaperone protein in the endoplasmic reticulum (ER) [75], acting through inositol-requiring enzyme 1α, which is a major stress sensor in the ER and regulates inflammatory cytokine production [76]. Immunomodulating properties of antidepressants in sepsis were also demonstrated for desipramine, fluoxetine, and amitriptyline, shown to decrease sepsis-induced organ damage [77, 78] and mortality [77] in murine sepsis models, through direct action on expression of inflammatory cytokines. Finally, in another study of murine sepsis, administration of trifuoroperazine, an antipsychotic agent, reduced organ damage and mortality through inhibition of pro-inflammatory cytokine surge, while reducing IL-10 levels [79]. These promising pre-clinical findings are yet to be examined in human sepsis trials. Our data set did not include information on use of psychotropic medications in patients with mental disorders. Further studies of more granular data are needed to examine the association of specific psychotropic therapy and sepsis-associated outcomes among patients with mental disorders.

### Study strengths and limitations

Our study has relevant strengths and limitations. In terms of strengths, the present study evaluates a little-examined and important research question and includes a large cohort of consecutive sepsis hospitalizations from an entire population. We adhered closely to reporting guidelines and used statistical methods to limit confounding and enhance trustworthiness in measures of association.

This study has, however, important limitations, in addition to those noted earlier, stemming mostly from use of administrative data and a retrospective design. First, although CCS codes for mental disorders were used in government reports [80] and prior epidemiological studies [81, 82], they were not validated and the optimal ICD-code-based algorithm to identify mental disorders in administrative data has not been determined. Thus, we cannot exclude misclassification between groups. However, misclassification of mental disorders would be expected to blur the differences between groups and thus diminish outcome differences between septic patients with and without mental disorders, leading to possible underestimation of the magnitude of the better outcomes observed among the former. Second, because we could not identify repeated admissions of individual patients in our cohort, it is possible that if the frequency of repeated admissions was higher in the group of patients with mental disorders than among those without these disorders, the denominator for calculation of short-term mortality would have been higher in the former. Such higher denominator could have resulted in erroneously lower estimate of short-term mortality among septic patients with mental disorders, which could not have been addressed on adjusted models. It should be noted, however, that similar methodological limitations affect epidemiological studies in the US based on other deidentified administrative data sets, including the National Inpatient Sample (NIS). However, in a recent study [83] based on the NIS data, that examined the prognostic impact of 3 autoimmune diseases in sepsis (where each of the studied autoimmune diseases is known to be associated with increased risk of sepsis and thus with increased risk of repeated hospital admissions related to sepsis compared to the general population, which could lead in turn to an increased likelihood of artificially lower apparent risk of death among the former, due to the considerations noted above), the risk-adjusted short-term mortality was either higher, lower, or not different among the patients with autoimmune diseases compared to the general population. Nevertheless, we cannot exclude the possibility that repeated hospital admission may have affected our outcome estimates. Third, we did not have information on the severity of mental disorders. In addition, our data set did not include information on processes of care, which may have differed between septic patients with and without mental disorders. Thus, we cannot exclude residual confounding in our models. It should be noted, however, that skewed severity of mental disorders to mostly mild disease would be expected to blur the outcome differences between the examined groups, while skewing toward mostly severe disease would not be expected to result in more favorable mortality outcomes among affected septic patients. In addition, as noted earlier, patients with mental disorders were reported to receive poorer quality of healthcare [10], and stigma, stereotyping, and negative attitudes towards these patients by clinicians have been previously documented [84, 85]. Such care differences would be not be expected, however, to result in better outcomes of septic patients with mental disorders. Fourth, our analyses may not be generalizable to categories of mental disorders not examined in the present study. Last, the generalizability of our findings to other countries and regions, beyond those previously studied, is unknown.

## Conclusions

Mental disorders were associated with lower risk of short-term mortality in sepsis. This favorable prognostic association was observed consistently for individual categories of mental disorders and on exploratory subgroup analyses. Additional studies to examine the factors underlying the conflicting across-country prognostic implications of mental disorders are needed to guide future efforts to improve sepsis outcomes.

## Supporting information

S1 File (DOCX)
