# Supplementary material for: Impact of history of mental disorders on short-term mortality among hospitalized patients with sepsis: A population-based cohort study
Source: PLoS One. 2022 Mar 10;17(3):e0265240. doi: 10.1371/journal.pone.0265240 (PMC8912146; doi:10.1371/journal.pone.0265240)
Supplement: S1 File — (DOCX) [file pone.0265240.s001.docx]

**Association of Mental Disorders with Short-Term Mortality in Sepsis: a Population-Based Cohort Study**

**Lavi Oud, M.D., John Garza, Ph.D.**

Supplementary Material

**S1 Table. International Classification of Diseases*,* Ninth and Tenth Revisions*,* Clinical Modification (ICD-9-CM and ICD-10-CM) codes used to identify procedures.**

**Variable ICD-9-CM ICD-10-CM codes**

Mechanical ventilation 96.70, 96.71, 96.72 5A1935Z, 5A1945Z, 5A1955Z

Hemodialysis 38.95, 39.95, V45.11, V56.0, V56.1 Z4901, 5A1D00Z, 5A1D60Z

Blood transfusion 99.00, 99.01, 99.02, 99.03, 99.04, 99.05 V58230233N1, 30243N1, 30253K1,30243K1

99.06, 99.07, 99.08, 99.09, V58.2 30253L1, 30243L1, 30233R1, 30243R1, 30240N0,  30240N1, 30230N0,   30230N1,  30240K0, 30240K1, 30240L0, 30240L1,  30240M0, 30240M1, 30230L0,  30230L1, 30230M0,  30230M1,  30240R0,  30240R1, 30230R0, 30230R1

**Methodology for assessment of the sensitivity of the association between mental disorders and short-term mortality in sepsis to missing gender data**

The state of Texas masks gender data of hospitalizations with a diagnosis of HIV infection, and of those with alcohol or substance use disorders. Gender data were missing nonrandomly in 8.8% of hospitalizations in our cohort. We examined the sensitivity of the association between mental disorders and short-term mortality in sepsis to missing gender data by using three different approaches:

1. Restriction of analyses to hospitalizations with reported gender data
2. Inclusion of all hospitalizations, with imputation of all missing gender data as “male” and then in a separate model as “female”
3. Inclusion of all hospitalizations with an indicator variable for those with missing gender data

The 3 approaches produced similar results (eTables 2-4 below). We present in the main manuscript the results of our analyses using data restricted to hospitalizations with gender data.

| **S2 Table.** **Multilevel multivariable logistic regression with propensity adjustment for the association**   \| **of mental disorders and short-term mortality: alternative modeling for the impact of missing gender** \| \|  \|  \| \| --- \| --- \| --- \| --- \| \|  \|  \|  \|  \| \| **Modeling approach** \| **aOR (95% CI)^a^** \| ***p* value** \|  \| \| Include only hospitalizations with gender data \| 0.7924 (0.7728-0.8125) \| <0.0001 \|  \| \| Assign all missing gender data as "male" \| 0.7863 (0.7678-0.8052) \| <0.0001 \|  \| \| Assign all missing gender data as "female" \| 0.7851 (0.7676-0.8040) \| <0.0001 \|  \| \| Include hospitalizations with missing gender data as indicator variable \| 0.7861(0.7676-0.8050) \| <0.0001 \|  \| \| a aOR (95% CI): adjusted odds ratio and 95% confidence intervals \| \| \| \| \|  \|  \|  \|  \| \|  \|  \|  \|  \| \|  \|  \|  \|  \| \| **S3 Table.** **Propensity matched cohort analysis of the association of mental disorders and short-term mortality:**   \| **alternative modeling for the impact of missing gender** \| \|  \|  \| \| --- \| --- \| --- \| --- \| \|  \|  \|  \|  \| \| **Modeling approach** \| **aOR (95% CI)^a^** \| ***p* value** \|  \| \| Include only hospitalizations with gender data \| 0.7968 (0.7688-0.8257) \| <0.0001 \|  \| \| Assign all missing gender data as "male" \| 0.7920 (0.7636-0.8215) \| <0.0001 \|  \| \| Assign all missing gender data as "female" \| 0.7933 (0.7650-0.8227) \| <0.0001 \|  \| \| Include hospitalizations with missing gender data as indicator variable \| 0.7896 (0.7614-0.8190) \| <0.0001 \|  \| \| a aOR (95% CI): adjusted odds ratio and 95% confidence intervals \| \| \| \|   **S4 Table.** **Multivariable logistic regression without propensity adjustment for the association** \| \|  \|  \| \| **of mental disorders and short-term mortality: alternative modeling for the impact of missing gender** \| \|  \|  \| \|  \|  \|  \|  \| \| **Modeling approach** \| **aOR (95% CI)^a^** \| ***p* value** \|  \| \| Include only hospitalizations with gender data \| 0.8062 (0.7865-0.8264) \| <0.0001 \|  \| \| Assign all missing gender data as "male" \| 0.7979 (0.7794-0.8169) \| <0.0001 \|  \| \| Assign all missing gender data as "female" \| 0.7969 (0.7784-0.8158) \| <0.0001 \|  \| \| Include hospitalizations with missing gender data as indicator variable \| 0.7979 (0.7794-0.8169) \| <0.0001 \|  \| \| a aOR (95% CI): adjusted odds ratio and 95% confidence intervals \| \| \| \| \|  \|  \|  \|  \| \|  \|  \|  \|  \| | | | | | | |  |  |  |  |  |
| --- | --- | --- | --- | --- | --- | --- | --- | --- | --- | --- | --- | --- | --- | --- | --- | --- | --- | --- | --- | --- | --- | --- | --- | --- | --- | --- | --- | --- | --- | --- | --- | --- | --- | --- | --- | --- | --- | --- | --- | --- | --- | --- | --- | --- | --- | --- | --- | --- | --- | --- | --- | --- | --- | --- | --- | --- | --- | --- | --- | --- | --- | --- | --- | --- | --- | --- | --- | --- | --- | --- | --- | --- | --- | --- | --- | --- | --- | --- | --- | --- | --- | --- | --- | --- | --- | --- | --- | --- | --- | --- | --- | --- | --- | --- | --- | --- | --- | --- | --- | --- | --- | --- | --- | --- | --- | --- | --- | --- | --- | --- | --- | --- | --- | --- | --- | --- | --- | --- | --- | --- | --- | --- | --- | --- | --- | --- | --- | --- | --- | --- | --- |
|  | | | | | |  |  |  |  |  |  |
| **S5 Table. The characteristics and outcome of propensity score matched septic hospitalizations** | | | | |  |  |  |  |  |  |  |
| **with and without mental disorders** | |  |  |  |  |  |  |  |  |  |  |
|  | |  |  |  |  |  |  |  |  |  |  |
|  | | **Mental disorders^a^** | **No mental disorders^a^** | **SD^b^** |  |  |  |  |  |  |  |
| **Variables** | | **n = 50,174** | **n = 50,174** |  |  |  |  |  |  |  |  |
| **Age, years** | |  |  | 0.0126 |  |  |  |  |  |  |  |
| 18-44 | | 5,058 (10.1) | 4,926 (9.8) |  |  |  |  |  |  |  |  |
| 45-64 | | 17,551 (35.0) | 17,568 (35.0) |  |  |  |  |  |  |  |  |
| ≥ 65 | | 27,565 (54.9) | 27,680 (55.2) |  |  |  |  |  |  |  |  |
| **Gender** | |  |  | 0.0009 |  |  |  |  |  |  |  |
| Female | | 30,734 (61.3) | 30,749 (61.3) |  |  |  |  |  |  |  |  |
| **Race/ethnicity** | |  |  | 0.0077 |  |  |  |  |  |  |  |
| White | | 30,641 (61.1) | 30,705 (61.2) |  |  |  |  |  |  |  |  |
| Hispanic | | 11,109 (22.1) | 10,984 (21.9) |  |  |  |  |  |  |  |  |
| Black | | 5,050 (10.1) | 5,030 (10.0) |  |  |  |  |  |  |  |  |
| Other | | 3,464 (6.9) | 3,455 (6.9) |  |  |  |  |  |  |  |  |
| **Health insurance** | |  |  | 0.0100 |  |  |  |  |  |  |  |
| Private | | 15,687 (31.3) | 15,780 (31.5) |  |  |  |  |  |  |  |  |
| Medicare | | 27,175 (54.2) | 27,184 (54.2) |  |  |  |  |  |  |  |  |
| Medicaid | | 4,025 (8.0) | 3,972 (7.9) |  |  |  |  |  |  |  |  |
| Uninsured | | 2,697 (5.4) | 2,672 (5.3) |  |  |  |  |  |  |  |  |
| Other | | 590 (1.2) | 566 (1.1) |  |  |  |  |  |  |  |  |
| **Deyo comorbidity index** | |  |  | 0.0094 |  |  |  |  |  |  |  |
| 0 | | 9,500 (18.9) | 9,500 (18.9) |  |  |  |  |  |  |  |  |
| 1-2 | | 19,415 (38.7) | 19,150 (38.2) |  |  |  |  |  |  |  |  |
| ≥ 3 | | 21,529 (42.9) | 21,524 (42.9) |  |  |  |  |  |  |  |  |
| **Major comorbidities** | |  |  |  |  |  |  |  |  |  |  |
| Chronic lung disease | | 16,792 (33.5) | 16,185 (32.3) | 0.0364 |  |  |  |  |  |  |  |
| Congestive heart failure | | 15,174 (30.2) | 15,195 (30.3) | 0.0013 |  |  |  |  |  |  |  |
| Cerebrovascular disease | | 4,284 (8.5) | 4,278 (8.5) | 0.0006 |  |  |  |  |  |  |  |
| Renal disease | | 15,174 (30.2) | 15,195 (30.3) | 0.0013 |  |  |  |  |  |  |  |
| Diabetes | | 19,793 (39.4) | 19,798 (39.5) | 0.0003 |  |  |  |  |  |  |  |
| Malignancy | | 6,135 (12.2) | 6,104 (12.2) | 0.0027 |  |  |  |  |  |  |  |
| Liver disease | | 4,200 (8.4) | 4,082 (8.1) | 0.0121 |  |  |  |  |  |  |  |
| **Hospital mortality** | | 12,744 (25.4) | 16,115 (32.1) | 0.2111 |  |  |  |  |  |  |  |
| a The parenthesized figures represent percents; Percentage figures may not add to 100 due to rounding | | | | |  |  |  |  |  |  |  |
| b SD: Standardized difference | |  |  |  |  |  |  |  |  |  |  |

|  |  |  |  | | |  |  | | | |  |
| --- | --- | --- | --- | --- | --- | --- | --- | --- | --- | --- | --- |
|  |  |  |  | | |  |  | | | |  |
|  | | | |  |  | | |  |  |  | |

S1 Figure. Histograms of propensity scores of sepsis hospitalizations with and without mental disorders


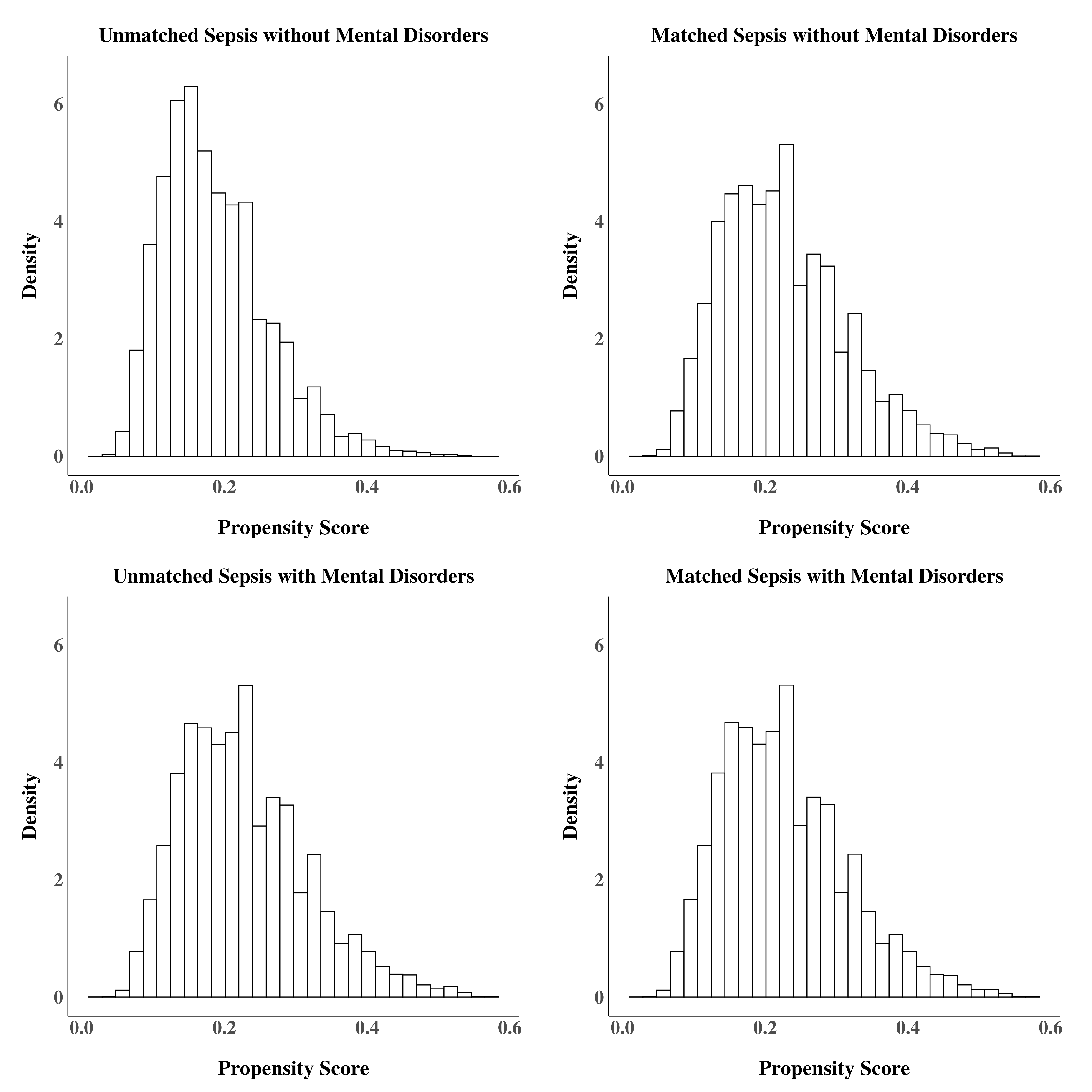


**R code used for study modeling**

# Load the MatchIt package
library(MatchIt)

# Apply matchit

m_out <-

matchit(

formula = comment ~

age + insurance + race_ethnic + gender +

deyoindex + deyolung + deyocvd + deyockd +

deyochf + dm + chrliver + malign +

comdrg1 + comalc1,

data = W,

method = 'nearest',

m.order = 'random',

ratio = 1,

replace = FALSE,

calcclosest = FALSE,

caliper = 0.2,

distance = 'logit')

*# Get matches form m_out*

Z <-

**get_matches**(

object = m_out,

model_frame = W,

id_cols = NULL,

newdata = NULL)

*# Define propensity scores*

propensity <- m_out$distance

# Load the lme4 package
library(lme4)

# Mixed-effects multivariable logistic regression with propensity adjustment
mllr_model <-

glmer(

formula = m_h ~

age + sex + race_ethnicity + insurance + deyo_index +

deyo_lung + deyo_cvd + dm + malign + oftotal +

year + procimvall + prochd + procbld + comment +

comdrg1 + comalc1 + propensity + (1|thcic_id),

data = M,

family = binomial(link = 'logit'),

control =

glmerControl(

optimizer = 'bobyqa',

optCtrl = list(maxfun = 1e6)),

start = NULL,

verbose = 0L,

nAGQ = 10,

contrasts = NULL,

devFunOnly = FALSE)

# Load the stats package
library(stats)

# Multivariable logistic regression without propensity adjustment
ordinary_model <-
 glm(
 data = M,
 formula = m_h ~
 age + sex + race_ethnicity + insurance + deyo_index +

deyo_lung + deyo_cvd + dm + malign + oftotal +

year + procimvall + prochd + procbld + comment +

comdrg1 + comalc1,

family = 'binomial')
